# Development of an Alcohol Dilution–Lyophilization Method for the Preparation of mRNA-LNPs with Improved Storage Stability

**DOI:** 10.3390/pharmaceutics15071819

**Published:** 2023-06-26

**Authors:** Daiki Shirane, Hiroki Tanaka, Yu Sakurai, Sakura Taneichi, Yuta Nakai, Kota Tange, Itsuko Ishii, Hidetaka Akita

**Affiliations:** 1 Laboratory of DDS Design and Drug Disposition, Graduate School of Pharmaceutical Sciences, Chiba University, 1-8-1 Inohana, Chuo-ku, Chiba 260-0856, Japan; ds.white.89@gmail.com; 2 Laboratory of DDS Design and Drug Disposition, Graduate School of Pharmaceutical Sciences, Tohoku University, 6-3 Aoba, Aramaki, Aoba-ku, Sendai 980-8578, Japan; yu.sakurai.e7@tohoku.ac.jp; 3 DDS Research Laboratory, NOF CORPORATION, 3-3 Chidori-cho, Kawasaki-ku, Kawasaki 210-0865, Japan; sakura_taneichi@nof.co.jp (S.T.); yuta_nakai@nof.co.jp (Y.N.); kota_tange@nof.co.jp (K.T.); 4 Department of Clinical Pharmacy, Graduate School of Pharmaceutical Sciences, Chiba University, 1-8-1 Inohana, Chuo-ku, Chiba 260-0856, Japan; iishii@faculty.chiba-u.jp

**Keywords:** lipid nanoparticle, mRNA delivery, freeze drying, storage stability

## Abstract

The lipid nanoparticle (LNP) is one of the promising nanotechnologies for the delivery of RNA molecules, such as small interfering RNA (siRNA) and messenger RNA (mRNA). A series of LNPs that contain an mRNA encoding the antigen protein of SARS-CoV-2 were already approved as RNA vaccines against this infectious disease. Since LNP formulations are generally metastable, their physicochemical properties are expected to shift toward a more stable state during the long-time storage of suspensions. The current mRNA vaccines are supplied in the form of frozen formulations with a cryoprotectant for preventing deterioration. They must be stored in a freezer at temperatures from −80 °C to −15 °C. It is thought that therapeutic applications of this mRNA-LNP technology could be accelerated if a new formulation that permits mRNA-LNPs to be stored under milder conditions were available. We previously reported on a one-pot method for producing siRNA-encapsulated LNPs by combining freeze-drying technology with the conventional alcohol dilution method (referred to herein as the “alcohol dilution–lyophilization method”). In this study, this method was applied to the preparation of mRNA-LNPs to provide a freeze-dried formulation of mRNA LNPs. The resulting formulation can be stored at 4 °C for at least 4 months.

## 1. Introduction

The lipid nanoparticle (LNP) is one of the most promising nanotechnologies for the delivery of RNA molecules, such as small interfering RNA (siRNA) and messenger RNA (mRNA), in both in vitro and in vivo situations. The key components of the LNPs are ionizable cationic lipids that contain pH-sensing tertiary amine groups in their structures [1]. The surface of LNPs containing ionizable cationic lipids had a neutral charge under physiological conditions. This neutral charge permits nonspecific electrostatic interaction with biomolecules to be avoided [2,3]. On the other hand, these ionizable cationic lipids develop a positive charge in an acidic environment. The positive charge facilitates the endosomal escape process via interactions with the anionic cellular membranes in the acidic endosomal compartment [4,5]. The usefulness of the LNPs has already been proven in the clinic. In 2018, ONPATTRO^®^, an LNP that contained a siRNA (siRNA-LNP) against transthyretin, was approved as the first siRNA therapeutic technology for the treatment of hereditary transthyretin-mediated amyloidosis [6]. Although the mainstream of the development of siRNA therapeutics is shifting to the use of ligand-conjugated siRNA, especially to the glycoprotein N-acetylgalactosamine (GalNAc)-conjugated siRNA for targeting the asialoglycoprotein receptor (ASGPR) on hepatocytes (i.e., Givlaari^®^, Oxlumo^®^, Leqivio^®^, and Amvuttra^®^), the LNP is still considered to be a key drug delivery system (DDS) technology for nucleic acid delivery [7].

LNPs have been shown to be important for the delivery of messenger RNA. Transfection with mRNA has been hampered by its high inflammatory properties. However, improvements in the quality of mRNA molecules (i.e., the introduction of chemically modified uridines [8,9], the addition of Cap1 structures [10], and the removal of dsRNA contaminants [11]) have made mRNA-based therapy a feasible modality. On the other hand, in the mRNA-based therapeutic approach, the high degradability of mRNA posed a problem since their phosphodiester linker cannot be chemically modified as siRNA. LNP technology has been used to protect the mRNA from degradation and to allow it to be delivered to the cytoplasm. Currently, a series of LNP-based RNA vaccines that contain an mRNA (mRNA-LNPs) encoding the mutated spike protein of SARS-CoV-2 were approved as a new modality for dealing with this infectious disease [12]. The use of LNP-based RNA therapeutics is expected to become a standard therapeutic approach in protein complementation therapy and regenerative medicine, as well as for use in vaccines for other infectious diseases or cancers [13].

Since it is generally assumed that the LNP is thermodynamically metastable, as in the case of a nanoemulsion [14,15], it is expected that physicochemical properties, such as particle size, surface charge, and polydispersity index (PdI), would change in a time-dependent manner. In the case of conventional liposomes that contain phospholipids, a subtransition from a tilted gel phase (Lβ′) to a crystalline phase (Lc) requires several days [16,17]. In the case of the Dipalmitoyl phosphatidylcholine liposome, the formation of an Lc phase required at least 6 days, and this transition continued to 28 days [18]. Therefore, during long-time storage, the intra-particle distribution of the lipid molecules in the LNP may change toward a more stable state, namely, an assembled structure of hydrophobic/amphiphilic molecules. It is also possible that the molecular structure of the components of the mRNA-LNPs would be changed via chemical reaction during storage. From the point of view of pharmaceutical products, the instability of physicochemical and/or chemical properties over time raises concerns about the deterioration of its function (i.e., protein expression and gene knockdown activity in mRNA-LNPs and siRNA-LNPs, respectively). The currently approved mRNA vaccines are distributed in the form of frozen formulations, and they must be stored at temperatures from −80 °C to −15 °C. This severe requirement regarding storage conditions is one of the major barriers to the global supply of RNA vaccines [19]. The non-vaccine use of the mRNA-LNPs, such as protein replacement therapy and cancer cytokine therapy, has also been extensively investigated [20]. Therefore, it is thought that the use of mRNA-LNPs could be expanded if a method for producing them would allow them to be stored under milder conditions.

For improving the storage stability of liposomal formulations, freeze drying (lyophilization) has been recognized as a useful method [19]. mRNA-LNPs are prepared by mixing an aqueous solution of the mRNA and an alcohol solution of the lipid to precipitate them into a complex. The process is followed by filtration to replace external solutions with buffers. For the lyophilization of mRNA-LNPs, cryoprotectant molecules, such as sucrose, are added to the suspension of mRNA-LNPs, and the resulting mixture is then freeze-dried. To simplify these formulation processes, we previously developed the “alcohol dilution–lyophilization method” for the preparation of siRNA-LNPs [21]. In this method, the solvent is simultaneously removed via freeze drying without the preliminary filtration. Thus, this method can be used to produce freeze-dried formulations directly from a mixture of nucleic acids and lipids as a one-pot process. In this study, the alcohol dilution–lyophilization method was used to prepare mRNA-LNPs based on a self-degradable ionizable lipid [22]. In the case of the previously reported siRNA-LNP, the mixture of siRNA and lipids was formulated by hand mixing. In this study, we used a microfluidic device for the mixing process with the goal of more accurately preparing LNPs. The resultant freeze-dried LNPs containing the mRNA can be stored at 4 °C (typical refrigerator conditions) for at least 4 months.

## 2. Materials and Methods

### 2.1. Materials

Detailed information on the suppliers of reagents used in this study, including item numbers of all reagents, is listed in Table S1 in Supplementary Materials. As a component of LNPs, a self-degradable lipid-like material was used (ssPalmO-Phe-P4C2) [22]. The ssPalmO-Phe-P4C2 (Product# COATSOME^®^ SS-OP), 1,2-dioleoyl-sn-glycero-3-phosphatidylcholine (DOPC, Product # COATSOME^®^ MC-8181), and 1-(Monomethoxy polyethyleneglycol2000)2,3-dimyristoylglycerol (DMG-PEG2000, Product # SUNBRIGHT^®^ GM-020) were supplied by NOF CORPORATION (Tokyo, Japan). Cholesterol was purchased from Sigma-Aldrich (St. Louis, MO, USA). The IVT-mRNA encoding reporter gene (firefly luciferase) was prepared via the in vitro transcription reaction described below and in Supplementary Materials. The mRNA encoding human Erythropoietin (hEPO) was purchased from TriLink BioTechnologies (San Diego, CA, USA). Quant-IT™ RiboGreen^®^ RNA reagent was purchased from Thermo Fisher Scientific (Waltham, MA, USA). All other reagents and chemicals were commercially available and were used without further purification.

### 2.2. Animals

BALB/c mice (females, 6 week-old) and C57BL/6J mice (females, 6 week-old) were purchased from Japan SLC, Inc. (Shizuoka, Japan). Protocols for the animal experiments were reviewed and approved by the Chiba University Animal Care Committee in accordance with the “Guide for Care and Use of Laboratory Animals”.

### 2.3. In Vitro Transcription to Prepare the IVT-mRNA

The pDNA-Luc was linearized by treatment with a restriction enzyme (AscI). mRNA-Luc was prepared according to the protocol for a MEGAscript™ T7 Transcription Kit (AM1334, Thermo Fisher Scientific, Waltham, MA, USA). The residual dsRNA was removed according to the article [23]. The 5′ Cap was added according to the protocol of ScriptCap Cap 1 Capping System (C-SCCS1710, Madison, WI, USA). The 3′ Poly(A) tail was added according to the protocol of the poly(A) Tailing Kit (AM1350, Thermo Fisher Scientific, Waltham, MA, USA). The detailed procedure for the in vitro transcription is summarized below.

#### 2.3.1. pDNA Linearization

A reaction mixture was prepared by mixing a 5 µg pDNA-Luc solution, 5 µL of Cut Smart buffer, and one µL of Restriction enzyme (AscI) and nuclease-free water was added to a volume of 50 µL. The mixture was incubated at 37 °C for 16 h. After adding 50 µL of nuclease-free water and 100 µL TE saturated phenol:CHCl_3_ = 1:1, the mixture was stirred for 1 min by vortex mixing. The mixture was centrifuged (4 °C, 15,000 rpm, 10 min), and the aqueous layer (upper layer) was collected. Nuclease-free water was added up to 100 µL; then 250 µL 99.5% EtOH, 5 µL 5M NaCl, 0.5 µL 20 mg/mL glycogen were added. The mixture was incubated in the −20 °C freezer for 15 min. After centrifuging (4 °C, 15,000 rpm, 15 min), the supernatant was removed. The pellet was washed by adding 200 µL of EtOH 70%. After centrifuging (4 °C, 15,000 rpm, 5 min), the supernatant was removed, then the pellet was dried for 3–5 min. The pellet dissolved in 20 µL nuclease-free water, and the concentration of DNA (linearized DNA) was then measured.

#### 2.3.2. In Vitro Transcription

mRNA-Luc was prepared according to the protocol of the MEGAscript™ T7 Transcription Kit (AM1334). The mixture was prepared by mixing 2 µL ATP solution (75 mM), 2 µL CTP solution (75 mM), 2 µL GTP solution (75 mM), 1.5 µL m1ΨTP (100 mM), 2 µL 10× Reaction buffer, and 1 µg linearized DNA, and adding nuclease-free water up to 20 µL. After 2 µL T7 Enzyme Mix was added, the mixture was incubated at 37 °C for 1 h. After 1 µL TURBO DNase was added, the mixture was incubated at 37 °C for 15 min. An amount of 30 µL nuclease-free water and 30 µL LiCl precipitation solution were added, and the suspension was incubated at −20 °C for 30 min. The mixture was centrifuged (4 °C, 20,400× *g*, 15 min). The supernatant was removed, and 200 µL EtOH 70% was added. The mixture was centrifuged (4 °C, 20,400× *g*, 5 min). The supernatant was removed, and the white pellet was dried for 30 s. The pellet was dissolved by adding 20 µL nuclease-free water and 480 µL chromatography buffer (10 mM HEPES, 0.1 mM 2NA(EDTA); 125 mM NaCl, 16% EtOH, pH 7.2) was then added.

#### 2.3.3. Cellulose Column Chromatography

The residual dsRNA was removed according to a previous article [23]. An amount of 0.2 g/mL cellulose suspension in chromatography buffer was prepared, and then the suspension was shaken at a high intensity (1200 rpm) for 10 min (in an incubator-shaker or vortex). After adding 350 µL of a cellulose suspension to the column, it was centrifuged (25 °C, 14,000× *g*, 60 s) by using a 90° microcentrifuge. An amount of 500 µL chromatography buffer was added into the column, followed by shaking for 5 min, at 25 °C, 1200 rpm. The column was centrifuged (14,000× *g*, 60 s) by using a 90° microcentrifuge. After adding 500 µL of mRNA solution to the column and shaking at 37 °C for 30 min (in a tube shaker), the column was centrifuged (14,000× *g*, 60 s) by using a 90° microcentrifuge. The solution was collected in a 1.5 mL tube, and 0.1 vol. (50 µL) of 3 M NaOAc, and 1 vol. (500 µL) isopropanol were then added. The mixture was incubated at −20 °C for 20 min, then centrifuged at 4 °C, 15,000× *g* for 10 min. The supernatant was removed, and the pellet was then washed by adding 200 µL 70% EtOH. The mixture was centrifuged at 4 °C, 20,400× *g*, and 5 min, after which the pellet was dissolved in 70 µL nuclease-free water.

#### 2.3.4. Capping

The 5′ Cap was added according to the protocol of ScriptCap Cap 1 Capping System (C-SCCS1710). An amount of 100 µg mRNA was taken from the 70 µL solution, then nuclease-free water was added up to 140 µL. The solution was incubated at 65 °C for 5–10 min, then placed on ice while preparing the capping reagents mixture. The mixture was prepared by mixing 20 µL 10× ScriptCap Capping Buffer, 20 µL 10 mM GTP, 5 µL 20 mM SAM, 5 µL ScriptGuard RNase Inhibitor, 8 µL ScriptCap 2′-O-Methyltransferase (100 U/µL), then 8 µL ScriptCap Capping Enzyme and 140 µL of mRNA solution were added to the mixture. The mixture was incubated at 37 °C for 30 min, and 100 µL LiCl precipitation solution was then added. The mixture was incubated at −20 °C for 30 min, then centrifuged (4 °C, 20,400× *g*, 15 min). The supernatant was removed, and the pellet was then washed by adding 200 µL EtOH 70%. After centrifuging (4 °C, 20,400× *g*, 5 min), the supernatant was removed, and the pellet was dried for 30 s. The resulting pellet was dissolved in 110 µL nuclease-free water.

#### 2.3.5. Poly(A) Tailing

The 3′ Poly(A) tail was added according to the protocol of the poly(A) Tailing Kit (AM1350). The mixture was prepared by mixing 110 µL mRNA solution, 40 µL 5× E-PAP Buffer, 20 µL 25 mM MnCl_2_, 20 µL ATP Solution, and 8 µL E-PAP Enzyme, then incubated at 37 °C for 45 min. After adding 100 µL LiCl precipitation solution, the mixture was then incubated at −20 °C for 30 min. After centrifuging (4 °C, 20,400× *g*, 15 min), the supernatant was removed, then the pellet was washed with 200 µL EtOH 70%. After centrifuging (4 °C, 20,400× *g*, 5 min), the supernatant was removed, then the pellet was dried for 30 s. The pellet was dissolved in 50 µL nuclease-free water and stored at −80 °C.

### 2.4. The Conventional Alcohol Dilution Method for mRNA-LNP (mRNA-LNPc)

The mRNA encoding luciferase was diluted with a 20 mM malic acid buffer (30 mM NaCl, pH 3.0) at a concentration of 0.0067 µg/µL. The lipid composition of the LNPs was ssPalmO-Phe-P4C2/DOPC/Cholesterol/DMG-PEG2000 in the following molar ratio: 52.5/7.5/40/1.5. The ethanol solution of the lipid was prepared at a concentration of 4 mM. These solutions were mixed using a microfluidics device (NanoAssemblr™, Precision NanoSystems, Vancouver, BC, Canada) (total flow rate; 4 mL/min, flow ratio; water/ethanol = 3/1 (*v*/*v*)). The mixture of the mRNA and lipids (0.8 mL) was recovered and diluted with 3 mL of MES/NaOH buffer (2-(N-morpholino) ethanesulfonic acid, 20 mM, pH 6.5). The external solution was replaced with phosphate-buffered saline without Ca^2+^ and Mg^2+^ (PBS(−)) by ultrafiltration using Amicon Ultra-4-100K centrifugal units. The particle solution was diluted to an adequate concentration with PBS(−) prior to transfection. mRNA-LNPs prepared via these conventional methods are referred to as mRNA-LNPc.

### 2.5. Alcohol Dilution–Lyophilization Method for mRNA-LNP (mRNA-LNPad)

The mRNA encoding luciferase or hEPO was diluted with a 20 mM malic acid buffer (pH 3.0) to a concentration of 0.0125 µg/µL. The lipid solution in 90%t-BuOH was prepared at a concentration of 20 mM. These solutions were mixed using a microfluidics device (total flow rate; fixed at 1 mL/min, flow rate ratios (FRR) in terms of water/t-BuOH (*v*/*v*) are indicated in each figure). The mixture of the mRNA and lipids (0.5 mL) was recovered, and an equivalent volume of sucrose solution was then added. The resulting mRNA-LNP suspensions containing sucrose were transferred to the shelf of a Drying Chamber DRC-1000 (EYELA, Tokyo, Japan), and the shelf was then cooled to −40 °C. The samples were lyophilized using a Freeze Dryer (FDU-1110; EYELA, Tokyo, Japan) that was connected to the Drying Chamber. Detailed settings of the segments (S1 to S9) were as follows: S1: −40 °C for 40 min; S2: −40 °C for 20 min (starting decompression); S3: −30 °C for 1 h; S4: −20 °C for 1 h; S5: −10 °C for 1 h; S6: 0 °C for 1 h; S7: 10 °C for 1 h; S8: 20 °C for 1 h, S9: 30 °C for at least 3 h until the end. Argon gas was then introduced into the chamber to stop the vacuum. The resulting lyophilized samples were resuspended in 500 µL of ultrapure water, and 500 µL of 2 × PBS was added under vortex mixing. The actual procedure for the particle preparation with images is shown in Figure S1. The temperature and pressure inside the chamber were measured using sensors attached to the apparatus and were monitored by a chart recorder μR-10000 (Yokogawa Electric Corporation, Tokyo, Japan). An example of the recorded chart showing temperature and pressure is shown in Figure S2. mRNA-LNPs prepared via this lyophilization method are referred to as mRNA-LNPad.

### 2.6. Characterization of the Particles

Size, Polydispersity Index (PdI), and Zeta potential of LNPs were measured with dynamic light scattering (Zetasizer Nano ZS, Malvern Panalytical, Malvern, UK). In these measurements, the particles were diluted with the 10 mM HEPES/NaOH buffer (pH 7.4), at least 8-fold. The encapsulation efficiency of the mRNA was evaluated by means of a Ribogreen^®^ assay. Ribogreen^®^ was diluted 200-fold in PBS with or without 0.4% *w*/*v* TritonX-100™. A calibration curve was prepared by sequential dilution from 0 to 2000 ng/mL of mRNA. The mRNA-LNP, corresponding to 50 ng of mRNA (in 50 µL), was mixed with an equal volume of the Ribogreen^®^ solution in a 96-well black plate. The plate was incubated for 5 min and shaken at 500 rpm in a shaking incubator. Fluorescence (Ex: 484, Em: 535) was evaluated by a plate reader (Infinite 200 PRO^®^; Tecan, Männedorf, Switzerland). The recovery ratio was calculated from total mRNA (with TritonX-100™) and mRNA input. Encapsulation efficiency was calculated from the concentration of mRNA that was not encapsulated (without TritonX-100™) and total mRNA.

### 2.7. Evaluation of Hepatic Gene Expression Efficiency (In Vivo Luciferase Assay)

A suspension of the LNP that contains messenger RNA encoding luciferase (mLuc-LNP) was diluted to the appropriate concentration with PBS. The mLuc-LNP suspension (5 µg mRNA/mL PBS) was administered to C57BL/6 mice via the tail vein. The injection volume was adjusted for body weight (10 µL/g). At 3 h post intravenous injection, the luciferase activity in the liver was measured. Details of the procedure used for the in vivo liver luciferase assay were reported in a previous study [24].

### 2.8. Evaluation of Hepatic Gene Expression Efficiency (IVIS Imaging Assay)

A suspension of mLuc-LNP was diluted to the appropriate concentration with PBS. The mLuc-LNP suspension (5 µg mRNA/mL PBS) was intravenously administered to the BALB/c mice via the tail vein at 10 µL/g (50 µg mRNA/kg body weight). At 3 h after the administration of the LNP, D-luciferin potassium PBS(−) solution (3 mg/200 µL/head) was injected intraperitoneally. At 5 min after the injection of the luciferin solution, the luminescence was measured with an In Vivo Imaging System (IVIS, Perkin Elmer, Waltham, MA, USA).

### 2.9. Human Erythropoietin ELISA Assay

The mhEPO-LNP was intravenously injected into the C57BL/6J mice via the tail vein. At 3, 6, and 24 h after the administration, blood samples were collected from the tail vein. Blood clotting was prevented by adding a heparin solution. Plasma was collected from the blood by centrifugation (4 °C, 2000× *g*, 20 min). The amount of hEPO in the plasma was measured by means of a Human Erythropoietin Quantikine ELISA Kit (R&D Systems, DEPRU0, Minneapolis, MN, USA).

### 2.10. Evaluation of Hepatotoxicity of LNP (AST/ALT ELISA Assay)

mRNA-LNPs were intravenously injected into mice via the tail vein at the dose indicated in the figure. At 3 h, 6 h, and 24 h after administration, blood samples were collected from the tail vein. After incubation of 2 h at room temperature, serum was collected from the blood by centrifugation (25 °C, 1000× *g* 20 min). The AST/ALT in serum was evaluated with an ALT Activity Assay Kit (MAK052, Merck, Rahway, NJ, USA) and AST Activity Assay Kit (MAK055, Merck).

## 3. Results

### 3.1. Strategy for the Alcohol Dilution–Lyophilization Method

**Figure 1:**
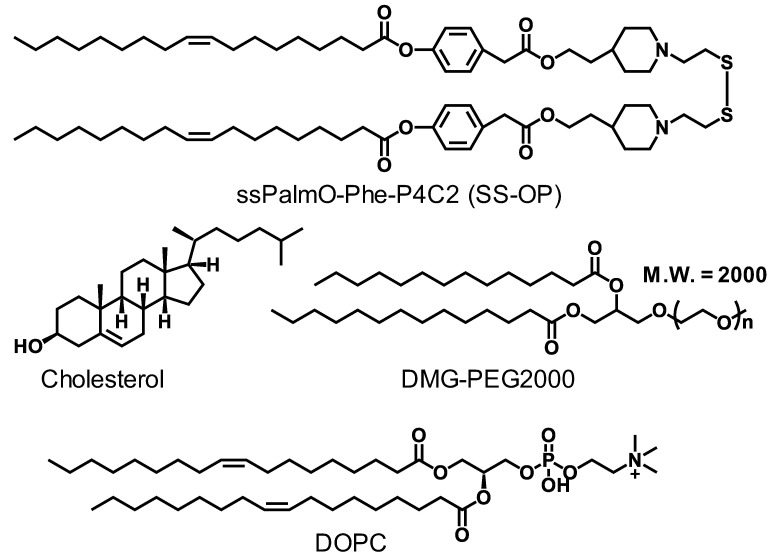
Chemical structures of lipid components. The structures of ssPalmO-Phe-P4C2, di-oleoyl-sn-glycero-phosphatidyl choline (DOPC), cholesterol, and polyethylene glycol-conjugated lipid (DMG-PEG2000) are shown.

An SS-cleavable and pH-activated lipid-like material (ssPalmO-Phe-P4C2) was used as the ionizable lipid (Figure 1). The ssPalmO-Phe-P4C2 contains a disulfide bond and phenyl esters, as well as the tertiary amine groups. The endosomal escape of the LNPs is facilitated by the tertiary amine groups. After the endosomal escape, the disulfide bond can be cleaved in a cytoplasm-selective manner since the intracellular concentration of glutathione, a natural reducing agent, is at least 1000-fold higher than the extracellular concentration. After the reductive cleavage, the thiol group attacks the phenyl ester groups. This hydrolytic reaction facilitates the intracellular release of the mRNA [22]. As additional components of the LNPs, di-oleoyl-sn-glycero-phosphatidyl choline (DOPC), cholesterol, and polyethylene glycol-conjugated lipid (PEG-lipid; DMG-PEG2000) were incorporated into the LNPs for stability (Figure 1). The composition of the LNPs was ssPalmO-Phe-P4C2/DOPC/cholesterol/PEG-lipid = 52.5/7.5/40/1.5 in molar ratio. It was reported that this composition allowed efficient mRNA delivery to the liver of a mouse when intravenously injected via the tail vein [22].

**Figure 2:**
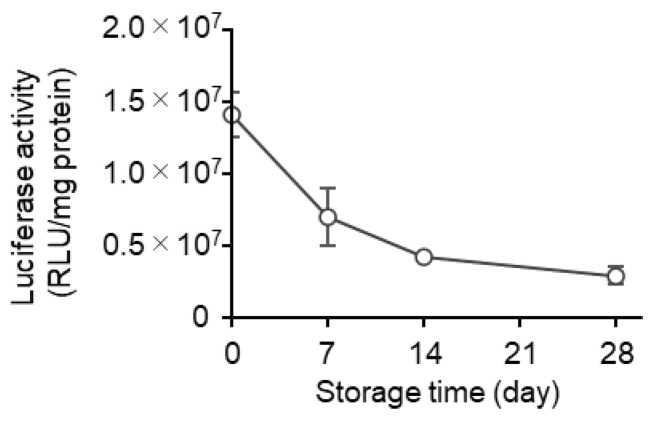
Time-dependent decrease in the activity of an mRNA-LNPc suspension. The mLuc-LNPc formulated by conventional microfluidic mixing was stored at 4 °C for periods from 0 to 28 days. The samples were injected into mice via the tail vein. At 3 h after the injection, the luciferase expression in the liver was evaluated. Each symbol indicates the Mean ± SD (n = 3).

At first, the LNPs were prepared via conventional microfluidic mixing (mRNA-LNPc), and the stability of this formulation at 4 °C in suspension form was evaluated. The conventional microfluidic mixing method involved the use of a NanoAssemblr™ microfluidic mixer. In this process, the pH of the mRNA solution needs to be acidic since the electrostatic interaction of the positively charged ionizable lipid in an acidic environment and the negatively charged mRNA molecules are a crucial driving force for the formation of the desired mRNA-LNPs. The external buffer was then replaced with PBS by ultrafiltration using Amicon Ultra centrifugal units. The resultant mRNA-LNPs showed an encapsulation efficiency of over 90% and were approximately 100 nm in size, with a PdI value below 0.1. The surface of the mRNA-LNPs was neutral since the Zeta potential was in the range of ±5 mV. The sample in PBS was stored at 4 °C for periods of up to 28 days (Table 1). Time-dependent changes in the particle properties and the gene expression efficiency were evaluated after 0, 7, 14, and 28 days (Figure 2). While changes in the particle properties (i.e., mRNA encapsulation efficiency, mRNA recovery efficiency, size, PdI, and zeta potential) were all negligible (Table 1), the gene expression efficiency was drastically reduced in a storage time-dependent manner (Figure 2). In the first 7 days, the gene expression efficiency decreased to approximately half (49%) of that for the original preparation. In the next 21 days, the gene expression efficiency decreased gradually but steadily, eventually reaching 21% of the original preparation. These data clearly confirm that the long-time storage of the LNPs in suspension form impairs their gene expression efficiency, while the changes in the physicochemical properties of particles are marginal.

**Figure 3:**
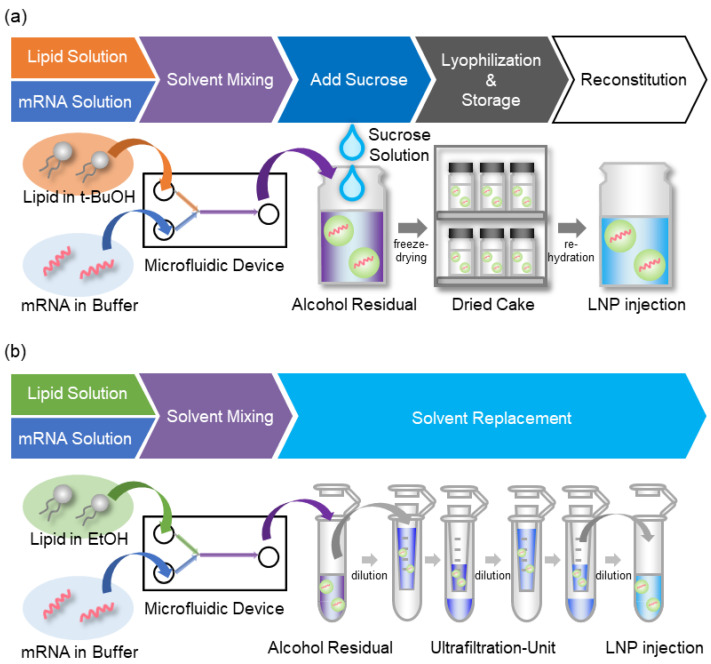
Schematic illustration of the alcohol dilution–lyophilization method and the conventional method. (**a**) The lipid nanoparticles with mRNA were first formulated by mixing a lipid mixture in tBuOH and mRNA in an acidic buffer. After the mixing, sucrose was added to the mRNA-LNP suspension as a cryoprotectant. The mixture was then freeze-dried to produce the dried formulation. (**b**) In the conventional method, particles are first formed using EtOH, and the solvent is then replaced using an ultrafiltration unit.

To develop a stable lyophilized formulation, mRNA-LNPs were prepared via the alcohol dilution–lyophilization method (mRNA-LNPad). A schematic illustration of the method is shown in Figure 3. In the case of conventional microfluidic mixing, the solvent for the lipid is ethanol. Since it is difficult to freeze ethanol because of its low melting temperature, the solvent for the lipids was replaced with tertiary butanol (tBuOH). The high melting temperature of tBuOH is suitable for the lyophilization process [25]. The LNP was first formulated by microfluidic mixing as the ethanol dilution method. After the mixing, sucrose was added to the LNPs as a cryo-protectant. The mixture was then freeze-dried to produce the actual lyophilized formulation (Figures S1 and S2). In the following sections, the effects of various parameters on the preparation were evaluated with the goal of establishing a method for preparing mRNA-LNPs in lyophilized form.

### 3.2. Effects of Flow Rate Ratio (FRR) of the Lipid/mRNA Solution and Sucrose Concentration

**Figure 4:**
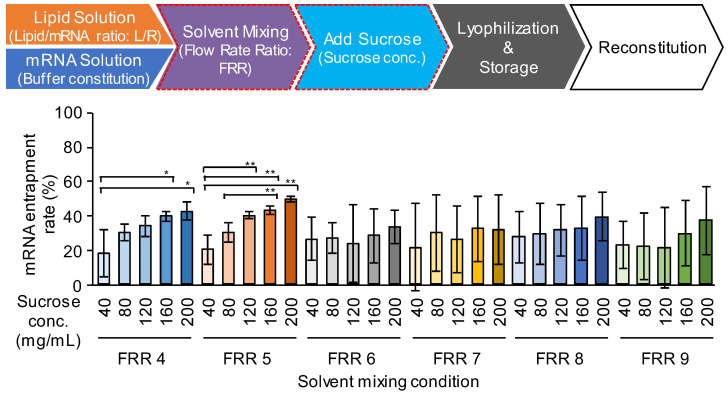
Effects of the sucrose concentration and the FRR in microfluidic mixing. The mRNA-LNPad was formulated via alcohol dilution–lyophilization method. The mRNA-LNPad was prepared for Lipid/mRNA ratio (nmol/μg) of 200. mRNA encapsulation efficiency of the particle prepared by indicated sucrose concentration and FRR condition was shown. The mRNA encapsulation efficiency was evaluated by a Ribogreen^®^ assay. Each bar indicates the Mean ± SD (n = 3). Statistical analyses of each FRR were performed by one-way ANOVA followed by the SNK-test. *: *p* < 0.05, **: *p* < 0.01.

The effects of the concentration of sucrose and the FRR were investigated from the point of view of mRNA encapsulation efficiency and the polydispersity index (PdI) of the mRNA-LNPad (Figure 4 and Figure S3). FRR is a parameter that dictates the volume ratio of the mRNA solution to the lipid solution: a high FRR is associated with the rapid dilution rate of the alcohol. The mRNA encapsulation efficiency was evaluated by means of a RiboGreen^®^ assay [24]. Since this RNA-binding fluorescent dye is not membrane permeable, the total mRNA and free mRNA can be distinguished in the presence or absence of a surfactant (Triton X-100™). The results indicated that the use of a high concentration of sucrose was beneficial for improving the mRNA encapsulation efficiency at FRR4 and FRR5. A high FRR above FRR6 resulted in poor reproducibility for the mRNA encapsulation efficiency (large batch-to-batch deviation). On the other hand, the PdI of the LNPs tended to increase in the presence of sucrose at a concentration of 200 mg/mL compared to 160 mg/mL at FRR4-7 (Figure S3a). In the hydration process of the formulation with this high level of sucrose, the dried cake did not dissolve uniformly since collapsed regions that were resistant to dissolution were formed. This observation suggests that when an excess amount of cryoprotectant was used, the primary drying was insufficient. This might cause the formulation to partially collapse and inhibit the further evaporation of water from the samples. This heterogeneous rehydration process could result in a formulation that contained heterogeneous LNPs with a large PdI. No trend in the particle size was observed among the ranges of the sucrose evaluated (Figure S3b). Since the LNPs with the smallest PdI value were prepared at an FRR4 with a sucrose concentration of 160 mg/mL, this condition was used in our further investigations.

### 3.3. The Effects of the Buffers and Lipid/mRNA Ratio

**Figure 5:**
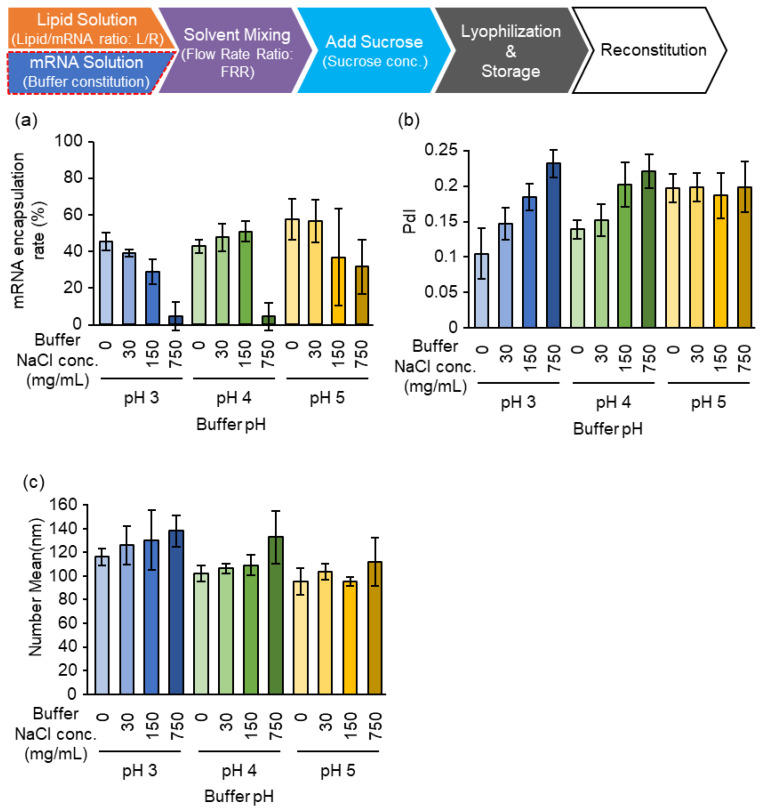
Effects of pH and salt concentration. The mRNA-LNPad was formulated via alcohol dilution–lyophilization method. The mRNA-LNPad was prepared for Lipid/mRNA ratio (nmol/μg) of 200. (**a**) mRNA encapsulation efficiency, (**b**) PdI, and (**c**) Size of the particle were prepared using the buffer at the indicated pH and salt concentrations. The size and PdI were evaluated by dynamic light scattering. The mRNA encapsulation efficiency was evaluated via Ribogreen^®^ assay. Each bar indicates Mean ± SD (n = 3).

The effects of the concentration of sodium chloride (NaCl) and the pH of the malic acid buffer in the solutes of mRNA were investigated. As shown in Figure 5, the effects of NaCl concentration depend on the pH of the buffer. At pH 3.0, an increase in the NaCl concentration results in a decrease in mRNA encapsulation efficiency and an increase in particle size and PdI. At pH 4.0, the encapsulation efficiency is drastically decreased at 750 mM NaCl, but the particle size and PdI showed a similar trend to the effects observed at pH 3.0. At pH 5.0, the encapsulation efficiency was decreased slightly in a NaCl concentration-dependent manner, while the particle size and PdI were not affected. Under these conditions, we concluded that the high salt concentration probably inhibits the interaction between the lipids and the mRNA. From the viewpoint of particle homogeneity, the PdI of the particles increased at higher pH. From these observations, we concluded that a pH of 3.0 and no additional salt in the malic acid buffer is required for preparing homogenous particles. The pH values of the sample before and after lyophilization were comparable (pH 3.2).

**Figure 6:**
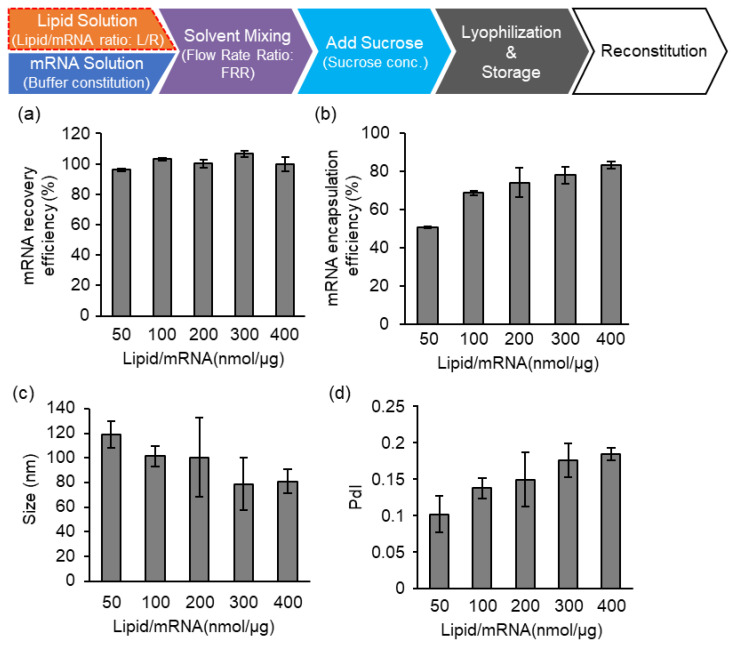
Effects of Lipid/mRNA ratio. The mRNA-LNPad was formulated via alcohol dilution–lyophilization method. (**a**) mRNA recovery efficiency and (**b**) mRNA encapsulation efficiency of particles prepared at the indicated Lipid/mRNA ratios (nmol/μg). The mRNA encapsulation efficiency was evaluated by a Ribogreen assay. (**c**) The size and (**d**) PdI were evaluated by dynamic light scattering. Each bar indicates Mean ± SD (n = 3).

Lastly, the effects of the lipid/mRNA ratio (nmol/μg) were evaluated (Figure 6). Since there is no buffer replacement process during which the materials included in the samples might be lost, the recovery efficiency of the mRNA against the initial input of the mRNA was 100% regardless of the lipid/mRNA ratio (Figure 6a). On the other hand, the encapsulation efficiency of the mRNA was improved in a lipid amount-dependent manner (Figure 6b). From these investigations, we fixed the parameters as follows: pH 3.0 malic acid buffer (without additional salt); FRR4; 160 mg/mL of sucrose; and a lipid/mRNA ratio of 400, which corresponds to the N/P ratio of 163. It should be noted that the ssPalm contains two amine moieties in one molecule. The effect of long-term storage was evaluated using the LNPs prepared under the above specifications.

### 3.4. Effects of Long-Term Storage on In Vivo Function

**Figure 7:**
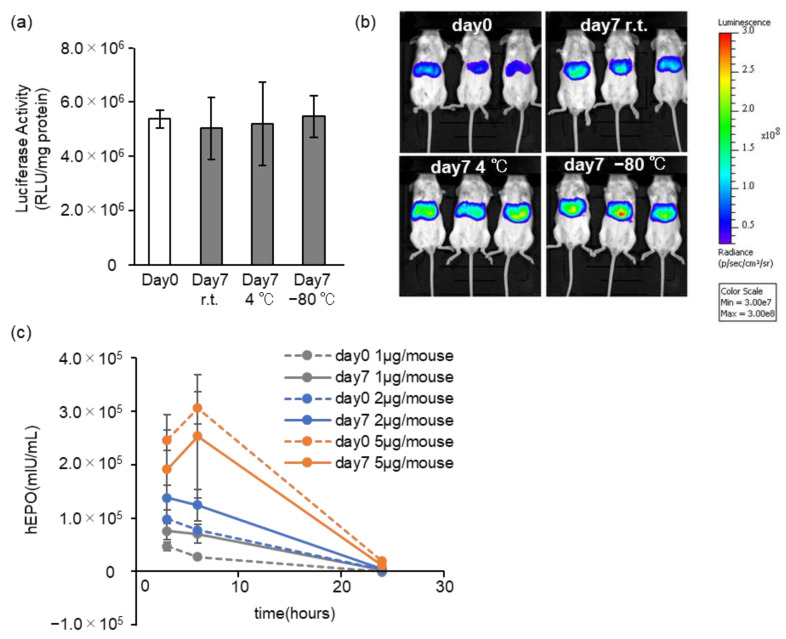
Effects of storage for first 7 days. The mRNA-LNPad was formulated via alcohol dilution–lyophilization method. These mRNA-LNPads were prepared for Lipid/mRNA ratio (nmol/μg) of 400. (**a**) Luciferase activity from the livers of mice. The Luc-LNPs stored at −80 °C, 4 °C, or r.t. (25 °C) for 7 days were injected into the mice via tail vein at 0.05 mg/kg. At 3 h after injection, the liver was collected, and the luciferase activity was evaluated (n = 3). (**b**) Visualization of the expression by an in vivo imaging system. At 3 h after intravenous injection, D-luciferin potassium (3 mg/head/200 μL) was injected intraperitoneally. After 10 min, the luminescence for the luciferase was visualized (n = 3). (**c**) Time-dependent expression of a secreted protein. The hEPO-LNPs, freshly prepared or stored at 4 °C for 7 days, were injected into the mice via tail vein. At 3, 6, and 24 h after injection, blood was collected from the tail vein. The concentration of the hEPO in the blood was evaluated by ELISA (n = 3).

**Figure 8:**
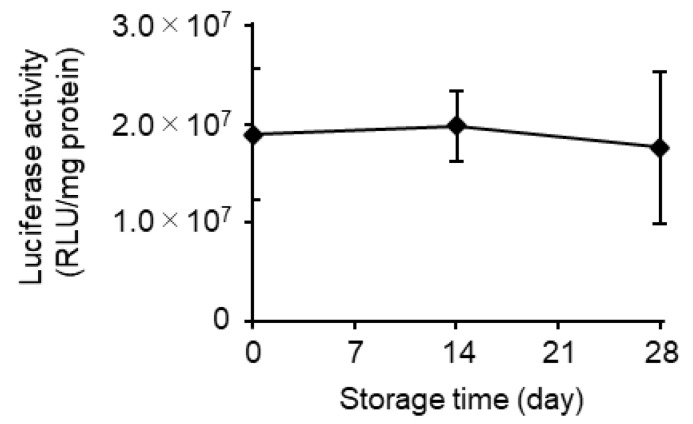
Storage stability analysis for 28 days. The mRNA-LNPad was formulated via alcohol dilution–lyophilization method. These mRNA-LNPad were prepared for Lipid/mRNA ratio (nmol/μg) of 400. The lyophilized samples, in freeze-dried form, were stored at 4 °C for 28 days. After rehydration, the mLuc-LNPad suspension (5 µg mRNA/mL) in PBS was then administered to mice via the tail vein. The injection volume was adjusted for body weight (10 µL/g). At 3 h post intravenous injection, the luciferase activity in the liver was evaluated (n = 3).

**Figure 9:**
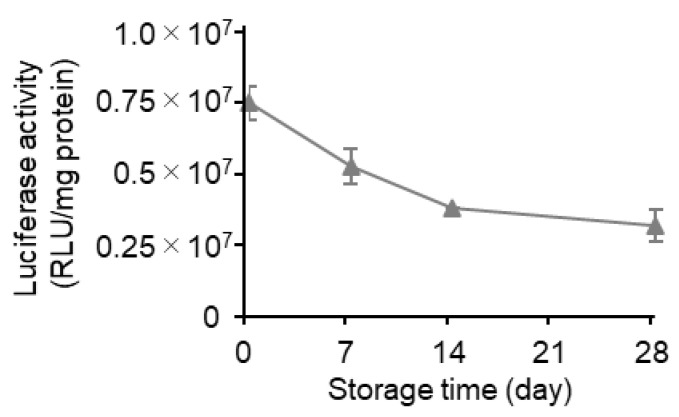
Time-dependent deterioration under atmospheric conditions. The mRNA-LNPad was formulated via alcohol dilution–lyophilization method. These mRNA-LNPads were prepared for Lipid/mRNA ratio (nmol/μg) of 400. At the time of release of the vacuum, the normal atmosphere was introduced to the dry chamber instead of argon gas. The resultant dried cake was stored at 4 °C for 4 weeks. At 0, 7, 14, and 28 days, the lyophilized samples were re-suspended in 500 µL of ultrapure water, and 500 µL of 2 × PBS was then added under vortex mixing. The rehydrated mLuc-LNPad suspension (5 µg mRNA/mL) in PBS was administered to mice via the tail vein. The injection volume was adjusted for body weight (10 µL/g). At 3 h post intravenous injection, the luciferase activity in the liver was evaluated (n = 2 (day14) or 3 (other time points).

**Figure 10:**
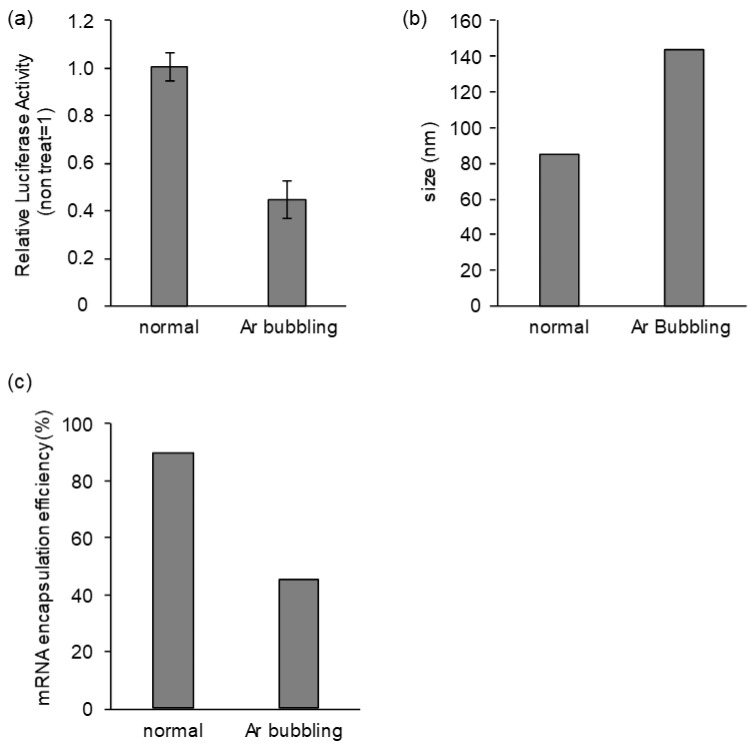
The effects of argon bubbling on the LNP suspension. Preparation of the mRNA-LNPc via the conventional alcohol dilution method as described in the main text. The argon bubbling treatment is conducted to the mRNA-LNPc suspension for 15 min via Pasteur pipette. The mRNA-LNPc suspension (5 µg mRNA/mL) in PBS was administered to mice via the tail vein. The injection volume was adjusted for body weight (10 µL/g). At 3 h post intravenous injection, (**a**) the luciferase activity in the liver was evaluated. (**b**) The particle size was evaluated by dynamic light scattering. (**c**) mRNA encapsulation efficiency of particles was evaluated by a Ribogreen assay.

The gene expression efficiency after storage was evaluated by the intravenous injection of mLuc-LNPad into mice. The storage conditions were room temperature (r.t.), 4 °C (refrigerator), and −80 °C (deep freezer). At 3 h after the injection, the protein expression of luciferase was quantitatively evaluated by means of a luminometer. At 7 days after the start of the storage, the gene expression efficiency was comparable to that for freshly prepared mRNA-LNPad regardless of the storage temperature (Figure 7a). The location of gene expression at 3 h after the injection was also evaluated by an in vivo imaging system (IVIS). The image of the IVIS experiments indicated that all of the formulation expressing the luciferase protein was predominantly in the liver (Figure 7b). The time-dependent expression of the protein human erythropoietin was also evaluated. At 3, 6, and 24 h after injection, blood was collected, and the concentration of the hEPO was evaluated by ELISA. The mRNA-LNPad that had been stored at 4 °C for 7 days was compared to the freshly prepared mRNA-LNPad (Figure 7c). The reduction in the expression was marginal even after 7 days of storage. The stability of the mRNA-LNPad that had been stored at 4 °C was further investigated for a period of 28 days. After storage for 14 days and 28 days, the gene expression efficiency of the freeze-dried formulation was unchanged (Figure 8). On the other hand, when rehydrated mRNA-LNPad from the same batch in suspension form was stored at pH 7.0 or pH 3.0 (Figure S4), the gene expression efficiency was reduced as the case for the mRNA-LNPc in suspension form prepared via conventional microfluidic mixing (Figure 2). These findings revealed that storage in the lyophilized form was important for maintaining the function of this mRNA-LNPad. The particle size and mRNA encapsulation efficiency were also maintained (Table 2). At the time of the release of the vacuum, the normal atmosphere was introduced to the dry chamber instead of argon gas, and the resultant dried cake was stored at 4 °C for 4 weeks (Figure 9). The activity of this mRNA-LNPad with atmosphere decreased by 29.3% at 7 days, 49.2% at 14 days, and 57.4% at 28 days. This finding suggests that the dried samples that had been exposed to the atmosphere were susceptible to time-dependent deterioration. To evaluate whether argon gas can improve the storage stability of mRNA-LNP suspension, mRNA-LNPc suspension prepared via the conventional alcohol dilution method was treated with argon bubbling for 15 min via Pasteur pipette. However, the mRNA-LNPc solution became cloudy by the bubbling of argon gas. The bubbling of argon gas increased the particle size by 68.3% and decreased the mRNA encapsulation efficiency by 49.7% (Figure 10). The gene expression activity was also reduced by 55.4% by the bubbling of argon gas.

**Figure 11:**
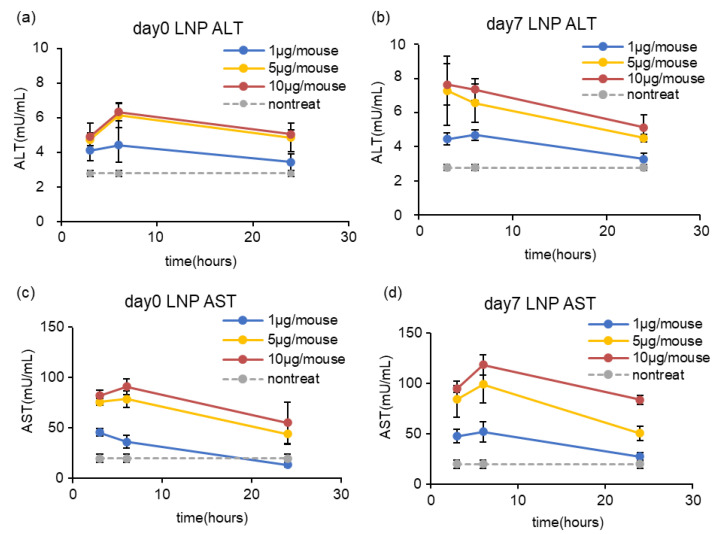
Liver toxicity of the formulation. The mRNA-LNPad was formulated via alcohol dilution–lyophilization method. These mRNA-LNPad were prepared for Lipid/mRNA ratio (nmol/μg) of 400. mRNA-LNPads that were reconstituted immediately after lyophilization or after storage for 7 days at 4 °C were intravenously injected into mice via tail vein at the dose indicated in the figures. At 3 h, 6 h, and 24 h after administration, blood samples were collected from the tail vein. After incubation of 2 h at room temperature, serum was collected from the blood by centrifugation (25 °C, 1000× *g* 20 min). The mouse serum ALT was evaluated with the ALT Activity Assay Kit after administration of particles stored for (**a**) 0 or (**b**) 7 days, respectively. The mouse serum AST was evaluated with the AST Activity Assay Kit after administration of particles stored for (**c**) 0 or (**d**) 7 days, respectively. Each bar indicates Mean ± SD (n = 3).

To evaluate the hepatotoxicity of mRNA-LNPad, the mRNA-LNPads that were reconstituted immediately after lyophilization or after storage for 7 days at 4 °C were intravenously injected into mice via tail vein at the dose indicated in Figure 11. At 3 h, 6 h, and 24 h after administration, the AST/ALT in serum was evaluated. At a dose of 1 µg/mouse, a slight increase in the ALT/AST concentration was observed at 3 h and 6 h after injection. At 24 h after injection, the ALT/AST concentration returned to the baseline. At a dose of 5 µg/mouse and 10 µg/mouse, the increase in the ALT/AST concentration was higher than that for the 1 µg/mouse. From 6 h to 24 h, the ALT/AST concentration tended to decrease (Figure 11).

**Figure 12:**
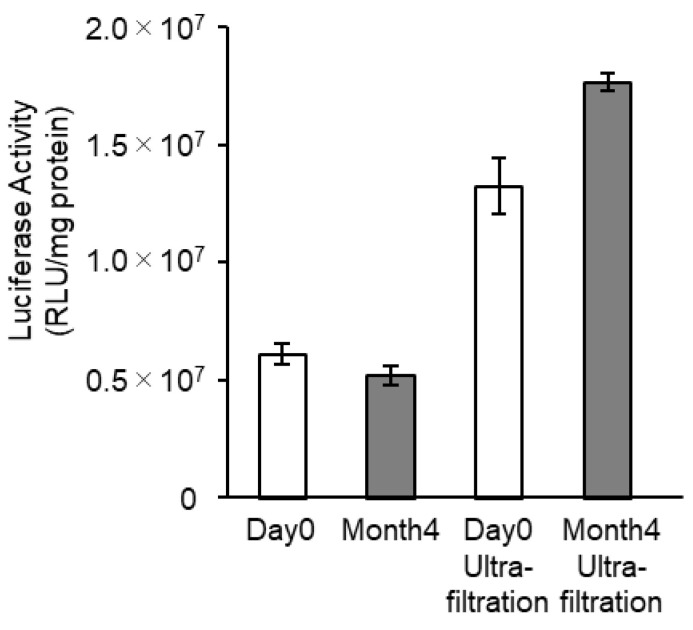
mRNA delivery efficiency after 4 months of storage. The mRNA-LNPad was formulated via alcohol dilution–lyophilization method. These mRNA-LNPad were prepared for Lipid/mRNA ratio (nmol/μg) of 400. The mRNA-LNPad was stored at 4 °C for 4 months. The samples were rehydrated and injected into the mice via the tail vein at a concentration of 0.05 mg/kg with or without ultrafiltration to remove cryoprotectant and buffer component. At 3 h after injection, the expression of the luciferase in the liver was evaluated. Each bar indicates the Mean ± SD (n = 3).

The in vivo mRNA transfection efficiency of the mRNA-LNPad was monitored after storage at 4 °C for 4 months (Figure 12). The lyophilized formulation of LNPs was rehydrated after storage and injected into the mice. Even after 4 months, the increase in the particle size was moderate, and their size was maintained at around 100 nm (Table 3). The encapsulation efficiency was also comparable to that of the freshly prepared LNPs. More importantly, the gene expression efficiency after storage for 4 months was comparable to that of the freshly prepared mRNA-LNPad.

## 4. Discussion

In this study, the alcohol dilution–lyophilization method was applied to the production of mRNA-LNPs to extend their stability in storage. Since the freeze-dried formulation prepared via the alcohol dilution–lyophilization method maintained its gene expression efficiency for at least 4 months at 4 °C, this formulation would be one of the preferable forms of mRNA-LNPs. Stabilization by freeze drying is more important for mRNA-LNPs than siRNA-LNPs since the mRNA-LNPs lose their gene transfection efficiency during the storage for a month (Figure 2), while the siRNA-LNP maintains its gene knockdown activity without lyophilization [21]

Zhao et al. evaluated the protein expression activity of lyophilized LNPs that were prepared using an ionizable lipid (TT3) after a long-term storage [26]. Consistent with our observations (Figure 2), the authors also found that the protein expression activity of the lipid-based nanoparticle system decreased to approximately 25% of that of the original preparation in 4 weeks. It was assumed that the functional half-life of the lipid-based nanoparticles encapsulating mRNA is approximately 1–2 weeks. It was also reported that the system lost its in vivo activity in 1 week, even in lyophilized form. In the case of RNA vaccines, it was reported that the production of antibodies against the receptor-binding domain of SARS-CoV-2 was not changed after the injection of LNPs that had been stored for at least 6 months in suspension form at 4 °C [27]. It was shown that there was no difference in the physicochemical properties of these LNPs. Luciferase activity and mRNA integrity were maintained at 4 °C by lyophilization in the presence of 10% sucrose and 10% maltose [28]. Suzuki et al. reported on the successful lyophilization of mRNA-LNPs containing L202, an ionizable lipid. The lyophilized mRNA-LNPs containing the SARS-CoV-2 protein induced antibody production against the antigen after 1-month storage at 25 °C. They reported that at least 4% sucrose was needed for a successful lyophilization [29]. These results indicated that, after long-term storage, the LNPs were capable of producing enough amounts of the antigen protein to activate acquired immunity. The lyophilized RNA vaccine can be used as a booster vaccine even in the case of vaccination for humans [30]. These results clearly suggest that these types of freeze-dried mRNA-LNPs can be useful. Since the continuous freeze drying [31] was applicable to the production of freeze-dried mRNA-LNPs [32,33], it would be possible to stably produce large quantities of such formulations.

An important issue for successful particle formation via the alcohol dilution–lyophilization method is that the freeze-drying process needs to be performed in a salt-free acidic buffer. Since the ssPalmO-Phe-P4C2 develops a positive charge in this acidic condition, the ssPalmO-Phe-P4C2 was able to interact with the mRNA during the rehydration process. The drastic decrease in the encapsulation efficiency of mRNA in the high concentration of NaCl can be explained by the shielding of these electrostatic interactions under the high ionic strength conditions (Figure 5a). It is noteworthy that the encapsulation efficiency of the mRNA-LNPc was also decreased in the case of a 750 mM NaCl concentration (Figure S5). This result indicates that the electrostatic interactions between nucleic acids and protonated ionizable lipids are perturbed in the presence of 750 mM NaCl in both cases. We concluded that the electrostatic interactions between mRNA and lipids, a crucial driving force for the formation of mRNA-LNPad, must be preserved during the manufacturing process of lyophilized LNPs. These observations suggest that the mRNA and lipid immediately after the alcohol dilution method form a complex via electrostatic interactions. However, it is possible that the organization of lipid molecules might not reach the final state to be a complete mRNA-LNPs. It is possible that the mRNA-LNPad is finally formed in the freeze-drying (removal of alcohol) and rehydration process. Further research is clearly needed to clarify the processes that are involved in the formation of this mRNA-LNPad complex.

Regarding the concentration of sucrose, the encapsulation efficiency and PdI of the particles varied depending on the sucrose concentration (Figure 4). Concerning encapsulation efficiency, the use of a higher sucrose concentration resulted in better encapsulation efficiency in FRR4 and FRR5. However, the use of a 200 mg/mL sucrose solution resulted in an increase in PdI in comparison with the 160 mg/mL in FRR4-FRR7. Therefore, 160 mg/mL was selected as the optimal concentration for improving the encapsulation ratio. The removal of the additive, cryoprotectants, and buffers used in the particle formation process by ultrafiltration was found to improve the gene expression efficiency approximately 2-fold (Figure 12 and Figure S6). One probable reason for this is that the mRNA-LNPad formulation after rehydration is hypertonic. Osmotic pressure should be carefully considered when considering the in vivo administration. In the case of ONPATTRO^®^, this formulation is diluted with a 0.9% NaCl solution to a total volume of 200 mL, filtered through a PES syringe filter, and then intravenously administered. Therefore, even when reconstituted mRNA-LNPad with a high sucrose solution is used, intravenous administration is possible if the preparation is first diluted with saline. Regarding intra-muscular injection, Comirnaty^®^ (0.45 mL of the formulation) is diluted (×5) to 1.8 mL with saline before the injection. If the freeze-dried formulation in this study was diluted similarly 5-fold, the calculated osmotic pressure ratio would be 1.16, thus making intramuscular injection also possible. Meanwhile, the effects of the cryoprotectant and buffer component on gene expression efficiency should be carefully evaluated.

A hypothesis concerning the decrease in the activity of mRNA-LNPs was recently reported [34]. In this report, the authors postulated that the N-oxidation of the ionizable lipids caused the production of aldehyde molecules. The aldehyde molecules then reacted with the mRNA encapsulated in the LNPs with the formation of mRNA-Lipid adducts, which would then inactivate the mRNA. At present, the molecular species that drive this N-oxidation of ionizable lipids are not clear. In the case of the peroxidation of polyunsaturated lipids that are formulated into liposomes, molecular oxygen dissolved in water was considered to be the cause of the oxidation reaction of hydrophobic chains [35,36]. Therefore, in this study, we postulated that the removal of oxygen species by eliminating the contact between particles with the water/atmosphere containing molecular oxygen would be important in terms of avoiding the lipid oxidation-mediated loss of function of LNPs and/or encapsulating mRNA. In this sense, the removal of the solvent for oxygen (water and alcohol) by lyophilization is a reasonable approach for long-term storage. Furthermore, the inclusion of an inert gas (argon or nitrogen) as a freeze-drying post-treatment may also be important. Actually, the lyophilized formulation prepared with including a normal atmosphere instead of argon gas loses its activity in a time-depending manner (Figure 9), although the freeze-dried formulation was still more stable than the mRNA-LNPc in suspension form (Figure 2). As another approach, it is possible that the direct bubbling of argon through a suspension of mRNA-LNPc could be used to remove the oxygen. However, this bubbling caused the severe aggregation of the LNPs and the leakage of mRNA from the particles. These events also resulted in a decrease in gene expression efficiency (Figure 10). Thus, the inclusion of an inert gas during the period with the pressure from the vacuum returned to atmospheric pressure appears to be a reasonable solution.

Our formulation is suspended in a hypertonic sucrose solution. The relative amount of lipid to the mRNA was also high. Therefore, it became important to evaluate the toxicity of the formulation. The formulation that was prepared via the alcohol dilution–lyophilization method caused slightly increased AST/ALT levels in the blood (Figure 11). However, the concentration of serum AST/ALT level was comparable to the LNPs that had been formulated using the microfluidic device [22]. Thus, it is unlikely that the method itself used to prepare the LNPs has adverse effects on biocompatibility.

## 5. Conclusions

In this study, we report on the successful use of the alcohol dilution–lyophilization method for preparing mRNA-LNPs. The key point in preparing the mRNA-LNPad is the condition of the buffers, such as the pH and salt concentration, while the key point for achieving storage stability is the lyophilization and storage in an inert gas, such as argon. An acidic pH and low salt concentration were found to be important in reconstituting LNP in the active form. Collectively, the formulation prepared via the alcohol dilution–lyophilization method expands the possibility of uses of mRNA-LNP since they can be stored at 4 °C for at least 4 months with their in vivo mRNA transfection function maintained.

## Figures and Tables

**Table 1:** Properties of particles prepared via conventional alcohol dilution method.

Storage (Days)	mRNA Recovery Efficiency (%) ^(a)^	mRNA Encapsulation Efficiency (%) ^(a)^	Size (nm) ^(b)^	PdI ^(b)^	Zeta Potential (mV) ^(b)^
0	96.5	88.9	102.2	0.066	−1.28
7	98.6	90.6	106.2	0.056	−2.34
14	90.2	86.9	100.2	0.079	−2.83
28	91.1	89.4	108.1	0.046	−3.45

^(a)^ mRNA recovery efficiency and mRNA encapsulation efficiency were evaluated by Ribogreen^®^ assay. ^(b)^ Size, polydispersity index (PdI), and Zeta potential were evaluated by dynamic light scattering.

**Table 2:** Properties of particles prepared via alcohol dilution–lyophilization method.

Storage (Days)	mRNA Recovery Efficiency (%) ^(a)^	mRNA Encapsulation Efficiency (%) ^(a)^	Size (nm) ^(b)^	PdI ^(b)^	Zeta Potential (mV) ^(b)^
0	103.9	77.2	78.5	0.202	2.03
7	105.5	76.3	73.5	0.198	1.59
14	104.3	80.9	70.0	0.183	−0.74
28	103.3	82.2	71.3	0.202	1.07

^(a)^ mRNA recovery efficiency and mRNA encapsulation efficiency were evaluated by Ribogreen^®^ assay. ^(b)^ Size, polydispersity index (PdI), and Zeta potential were evaluated by dynamic light scattering.

**Table 3:** Properties of particles before and after 4 months storage.

Samples	Storage Time (Months)	mRNA Encapsulation Efficiency (%) ^(a)^	Size (nm) ^(b)^	PdI ^(b)^	Z-Potential (mV) ^(b)^
After Reconstitution	0	60.1	75.1	0.177	−3.78
	4	68.1	106.8	0.192	−2.60
Reconstitution and Ultrafiltration	0	67.2	124.6	0.154	−3.46
	4	67.4	111.9	0.190	−2.91

^(a)^ mRNA encapsulation efficiency was evaluated by Ribogreen^®^ assay. ^(b)^ Size, polydispersity index (PdI), and Zeta potential were evaluated by dynamic light scattering.

## Data Availability

The data that support the findings of this study are available from the corresponding author, H.T. and H.A., upon reasonable request.

## Supplementary Materials

The following supporting information can be downloaded at: https://www.mdpi.com/article/10.3390/pharmaceutics15071819/s1, Table S1: Detailed list of information on suppliers of the reagents; Figure S1: Actual procedure for the particle preparation with images; Figure S2: Record of temperature and pressure during lyophilization; Figure S3: Dependency of the PdI and size on sucrose concentration; Figure S4: Storage stability analysis for 28 days; Figure S5: Effects of salt on conventional microfluidic mixing; Figure S6: Effects of removal of cryoprotectant and buffer component.
