# Supplementary material for: Development of an Alcohol Dilution–Lyophilization Method for the Preparation of mRNA-LNPs with Improved Storage Stability
Source: Pharmaceutics. 2023 Jun 26;15(7):1819. doi: 10.3390/pharmaceutics15071819 (PMC10383539; doi:10.3390/pharmaceutics15071819)
Supplement: Supplementary file 1 [file pharmaceutics-15-01819-s001.zip › pharmaceutics-2439255-supplementary.pdf]

Supplementary Materials

Title:

Development of an Alcohol Dilution–Lyophilization Method for the Preparation of mRNA-LNPs with Improved Storage Stability.

Author:

Daiki Shirane[1#], Hiroki Tanaka[2#*], Yu Sakurai[2], Sakura Taneichi[3], Yuta Nakai[3], Kota Tange[3], Itsuko Ishii[4], Hidetaka Akita[2*]

Affiliations:

[1] Laboratory of DDS Design and Drug Disposition, Graduate School of Pharmaceutical Sciences, Chiba University, 1-8-1 Inohana, Chuo-ku, Chiba 260-0856, Japan

[2] Laboratory of DDS Design and Drug Disposition, Graduate School of Pharmaceutical Sciences, Tohoku University, 6-3 Aoba, Aramaki, Aoba-ku, Sendai 980-8578, Japan

[3] DDS Research Laboratory, NOF CORPORATION, 3-3 Chidori-cho, Kawasaki-ku, Kawasaki 210-0865, Japan

[4] Department of Clinical Pharmacy, Graduate School of Pharmaceutical Sciences, Chiba University, 1-8-1 Inohana, Chuo-ku, Chiba 260-0856, Japan

[#] These authors equally contributed to this study.

* Corresponding Author:

e-mail address: hiroki.tanaka.e1@tohoku.ac.jp (H. Tanaka)

Tel.: +8143-226-2894

e-mail address: hidetaka.akita.a4@tohoku.ac.jp (H. Akita)

Tel.: ++8122-795-6831

TABLE OF CONTENTS

Supplementary Figures

S1. Supplementary tables

Table S1. Detailed list of information on suppliers of reagents

S2. Supplementary figures

S2-1 Figure S1. Actual procedure for the particle preparation with images

S2-2 Figure S2. Record of temperature and pressure during lyophilization

S2-3 Figure S3. Dependency of the PdI and size on sucrose concentration

S2-4 Figure S4. Storage stability analysis for 28 days

S2-5 Figure S5. Effects of salt on conventional microfluidic mixing

S2-6 Figure S6. Effects of removal of cryoprotectant and buffer component

Table S1. Detailed list of information on suppliers of the reagents

Reagent	Size	Manufacturer	Product number
COATSOME® SS-OP (ssPalmO-Phe-PrC2)	1 g	NOF CORPORATION	
Ethanol 99.5%	500 mL	Nacalai tesque	14712-05
t-Butyl Alcohol	500 mL	Nacalai tesque	11714-75
SUNBRIGHT® GM-020(DMG-PEG)	1 g	NOF CORPORATION	GM-020
COATSOME® MC-8181 (DOPC)	1 g	NOF CORPORATION	MC-8181
Cholesterol Sigma Grade, ≥99%	5 g	SIGMA Aldrich	C8667-5G
MES	100 g	Nacalai tesque	02442-44
DL-Malic Acid	500 g	Nacalai tesque	21029-55
Sodium Chloride	500 g	Nacalai tesque	31320-05
HEPES	500 g	DOJINDO LABORATORIES	342-01375
Sucrose	500 g	Nacalai tesque	30404-45
UltraPure™ DNase/RNase-Free Distilled Water	500 mL	Invitrogen™	10977023
D-PBS(-)	500 mL	Nacalai tesque	14249-24
Quant-iT™ RiboGreen® RNA reagent	1 mL	Invitrogen™	R11491
CleanCap® hEPO mRNA (ψU)	100 µg	TriLink BioTechnologies	Custom order
D-Luciferin Potassium Salt	1 g	FUJIFILM Wako Pure Chemical Corporation	126-05116
Luciferin Detection Reagent	10 mL	Promega	V8920
Tris-HCl, Molecular Biology Grade	100 g	Promega	H5121
EDTA・2Na	500 g	DOJINDO	345-01865
TritonX-100 ™	500 mL	Nacalai tesque	12969-25
TaKaRa BCA Protein Assay Kit	500 reaction	Takara Bio	T9300A
Heparin Sodium Injection 5,000 units/5mL MOCHIDA	5 mL	MOCHIDA PHARMACEUTICAL CO.,LTD.	224122458
AscI	500 units	New England Biolabs Japan	R0558S
Phenol:Chloroform:Isoamyl Alcohol 25:24:1 Mixed, pH 7.9	100 mL	Nacalai tesque	25970-14
MEGAscript™ T7 Transcription Kit	40 reactions	invitrogen	AM1334
ScriptCap Cap 1 Capping System	10 reactions	Cellscript	C-SCCS1710

poly(A) Tailing Kit	25 reactions	ThermoFisher Scientific	AM1350
N1-Methylpseudouridine-5'-Triphosphate	5 µmol	TriLink	N-1081-5
Glycogen Solution(20mg/ml) from Oyster, Nuclease tested	1 mL	Nacalai tesque	17110-11
Cellulose	100 g	Sigma-Aldrich	C6288-100G
Acetic Acid Sodium Salt	500 g	Nacalai tesque	09584-55
2-Propanol	500 mL	Nacalai tesque	29112-05
Human Erythropoietin Quantikine ELISA Kit, RU0	96 tests	R&D Systems	DEPRU0
ALT Activity Assay Kit	100 tests	Merck	MAK052
AST Activity Assay Kit	100 tests	Merck	MAK055

S2-1 Figure S1. Actual procedure of the particle preparation with images

The process of the alcohol dilution-lyophilization method is shown with images. The samples prepared by microfluidic mixing were then lyophilized as described in the Materials and Methods section. The mRNA-LNPad formulation can be used by hydrating with water.

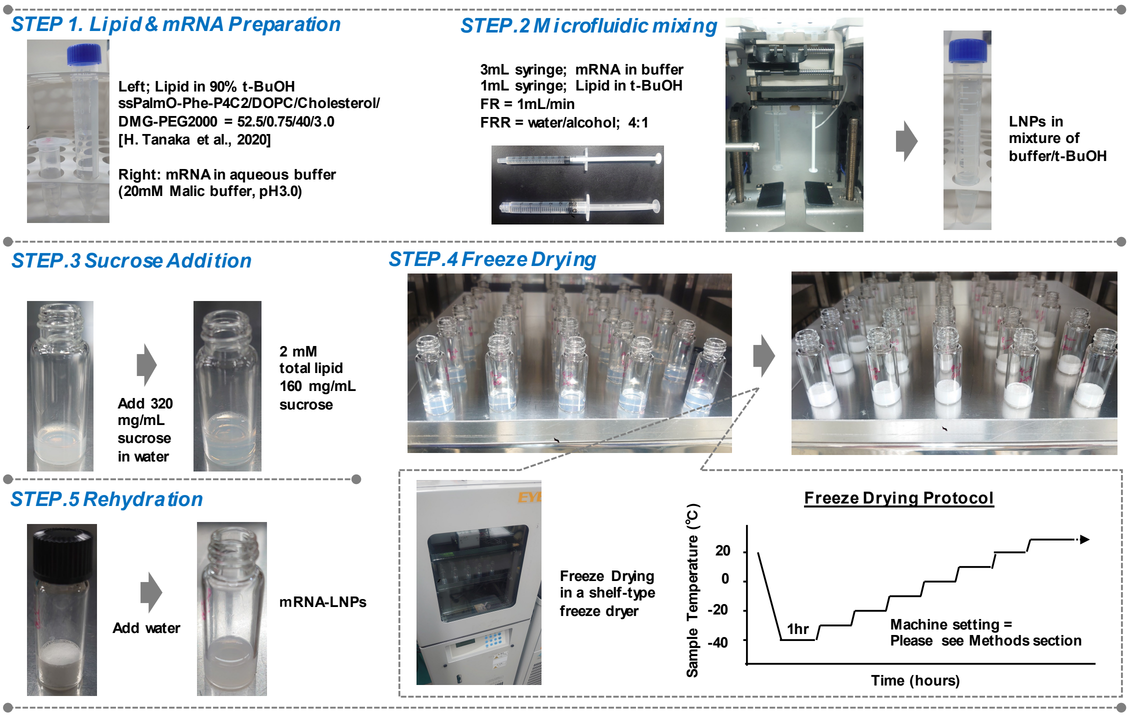

S2-2 Figure S2. Record of temperature and pressure during lyophilization

Suspensions of mRNA-LNPs containing sucrose were transferred to the shelf of a Drying Chamber DRC-1000 (EYELA, Tokyo, Japan). The samples were lyophilized using a Freeze Dryer (FDU-1110; EYELA, Tokyo Japan) that was connected to the Drying Chamber. Temperature and pressure inside of the chamber were measured using sensors attached to the apparatus and were monitored by a chart recorder μR-10000 (Yokogawa Electric Corporation, Tokyo, Japan). An example of such a chart is shown below.

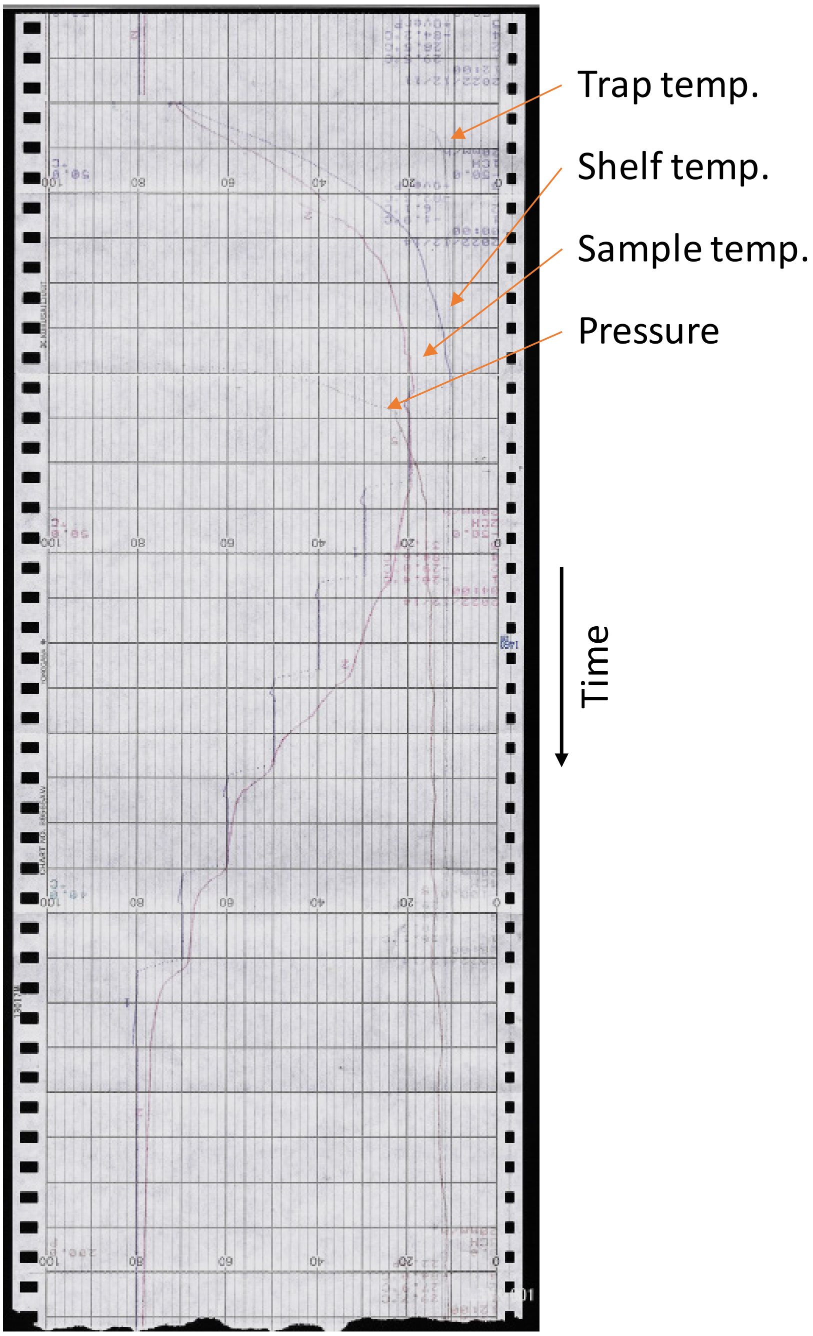

S2-3 Figure S3. Sucrose concentration dependency of the PdI and size

The mRNA-LNPad was formulated by alcohol dilution-lyophilization method. The mRNA-LNPad was prepared for Lipid/mRNA ratio (nmol/μg) of 200. Effects of the sucrose concentration and FRR in microfluidic mixing was investigated. a) PdI, and b) Size of the particle prepared by indicated sucrose concentration and FRR condition was shown. The size and PdI were evaluated by dynamic light scattering. Each bar indicates the Mean ± SD (n=3). Statistical analyses in the each FRR was conducted one-way ANOVA followed by SNK-test. *; $p < 0.05$, **; $p < 0.01$

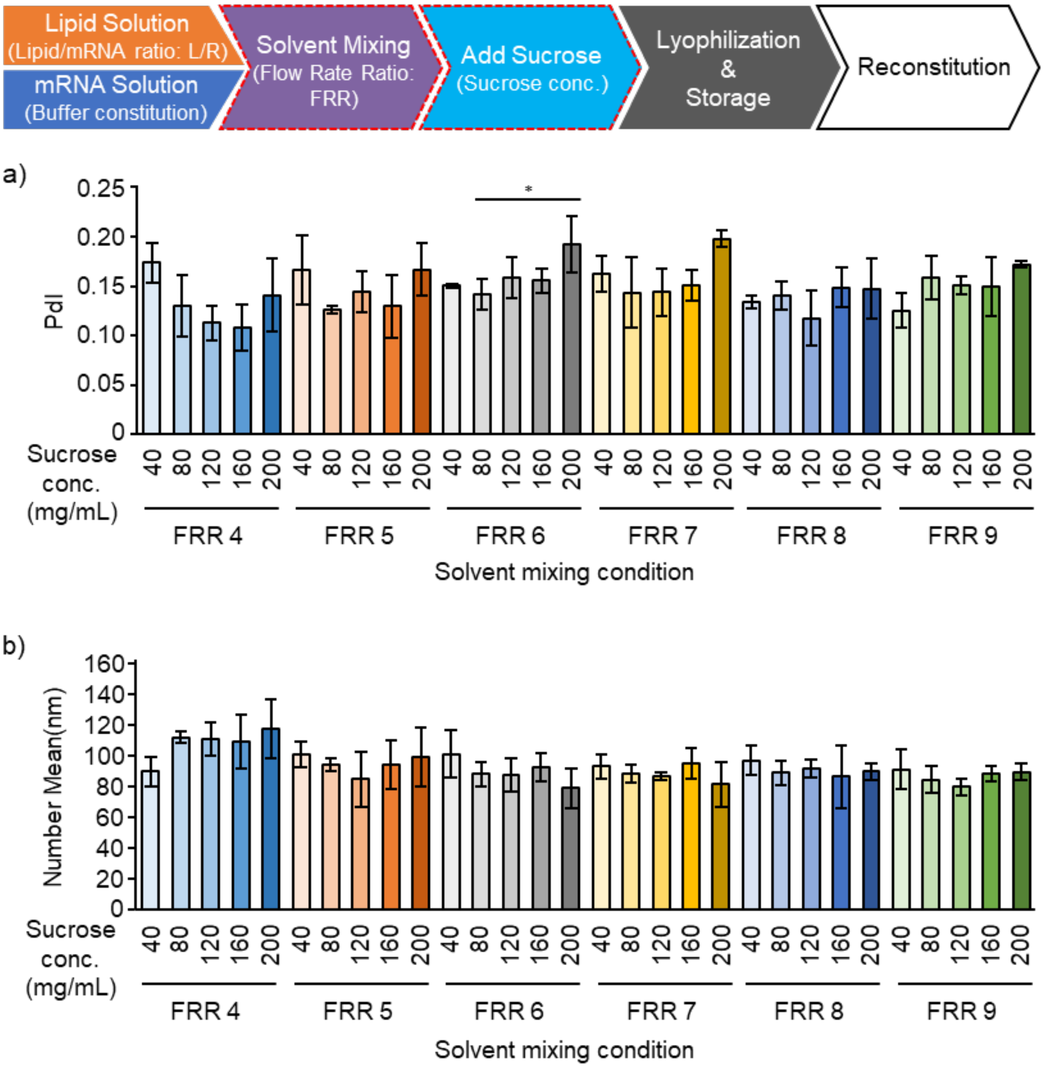

PdI values of the particles in a 200 mg/mL sucrose solution were larger than that at 160 mg/mL at FRR4-7. This observation indicates that an excess amount of sucrose might negatively affect the formulation. In the case of the size, no trend against the sucrose concentration and FRR was observed.

S2-4 Figure S4. Storage stability analysis for 28 days

The mRNA-LNPad was formulated by alcohol dilution-lyophilization method. The mRNA-LNPad was prepared for Lipid/mRNA ratio (nmol/μg) of 400. The lyophilized samples were rehydrated with 500 μL of water and an equal volume of 2×PBS was then added. The rehydrated samples, as well as the lyophilized samples in freeze-dried form, were stored for 28 days. The solution of these mRNA-LNPad samples in suspension form were replaced to PBS (pH7.4) or malic acid buffer (pH3.0) by ultrafiltration. To exclude the effects of buffer, all of the samples were ultrafiltrated again and the solution was replaced to PBS before injection. The mLuc-LNPad suspension (5 μg mRNA/mL) in PBS was then administered to mice (C57BL/6L mouse, female, 6 weeks; Japan SLC, Inc.) via the tail vein. The injection volume was adjusted for body weight (10 μL/g). the luciferase activity in the liver was evaluated At 3 hours post intravenous injection. Relative luciferase activity compared to freshly prepared one is plotted. (n=3)

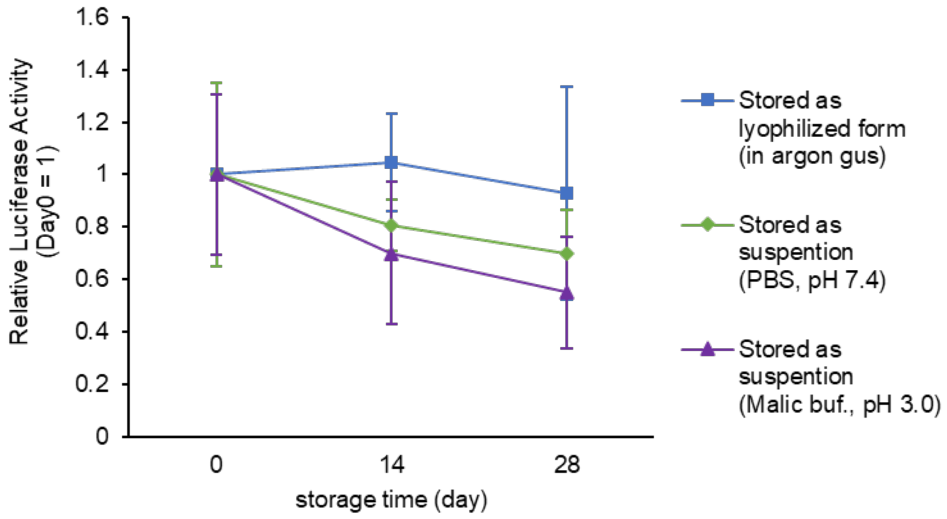

After storage for 14 days and 28 days, the gene expression efficiency of the mLuc-LNPad was retained when they were stocked in lyophilized form. On the other hand, when these lyophilized mLuc-LNPad were rehydrated, the gene expression efficiency was reduced as in the case of the LNPs in suspension form prepared by conventional microfluidic mixing (**Figure 2** in the main text) regardless of the storage pH (pH7.0 or pH3.0). Therefore, it was revealed that storage as the lyophilized form of the mRNA-LNPs are important for maintaining their function.

S2-5 Figure S5. Effects of salt on conventional microfluidic mixing

The mRNA-LNPc was prepared by conventional microfluidic mixing. The effects of salt concentration in the malic acid buffer on mRNA encapsulation, PdI, and size were investigated. The size and PdI were evaluated by dynamic light scattering. The mRNA encapsulation efficiency was evaluated by a Ribogreen® assay.

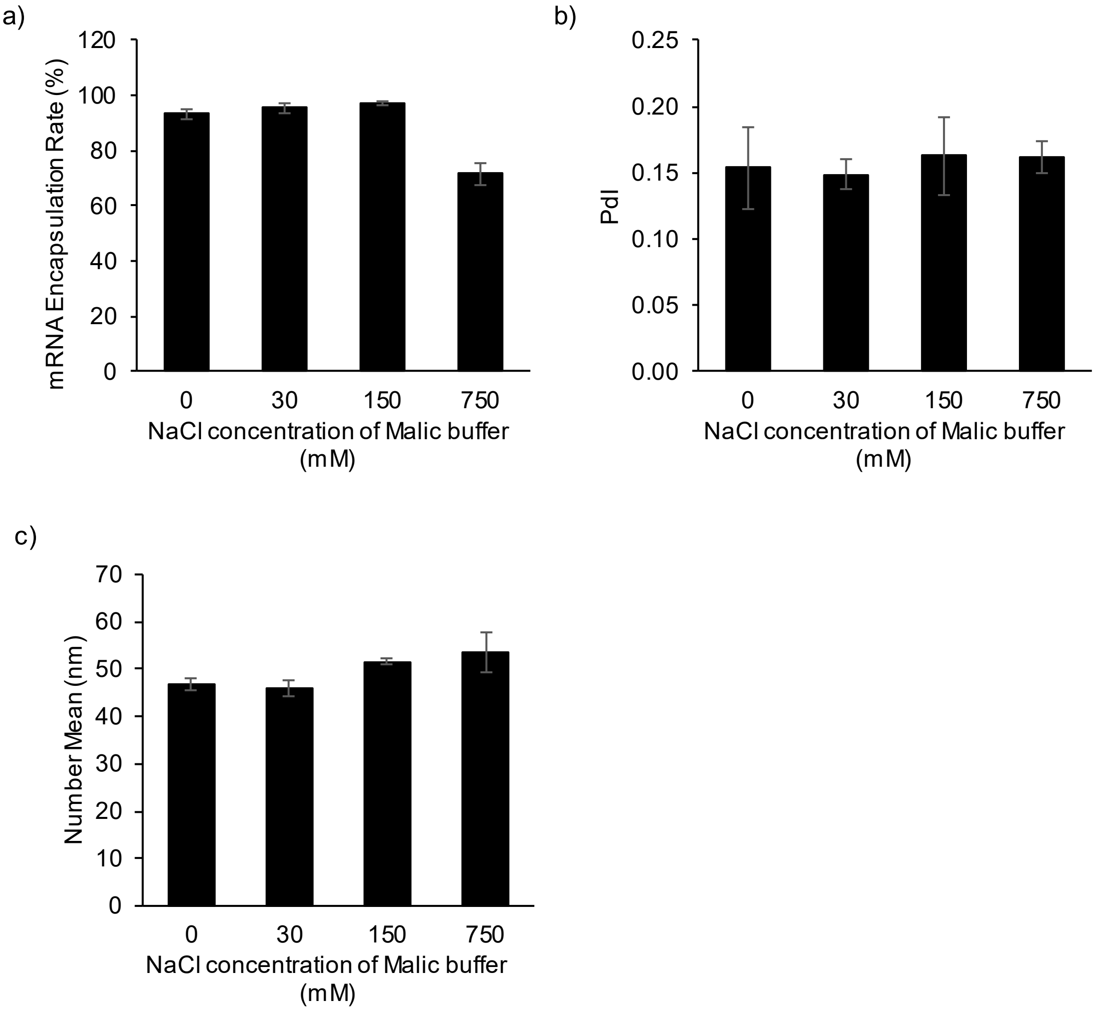

The encapsulation efficiency of the mRNA-LNPc prepared by conventional microfluidic mixing was decreased in the case when the 750 mM NaCl was used. No changes were observed for the size and PdI in this range. These data indicated that the electrostatic interaction between nucleic acids and protonated ionizable lipids was perturbed in the presence of 750 mM NaCl. This observation is consistent with the results shown in **Figure 5** in the main text.

S2-6 Figure S6. Effects of removal of cryoprotectant and buffer component

The mRNA-LNPad was formulated by alcohol dilution-lyophilization method. The mRNA-LNPad was prepared for Lipid/mRNA ratio (nmol/μg) of 400. The lyophilized samples were re-suspended in 500 μL of ultrapure water and neutralized with 500 μL of 2×PBS under vortex mixing. The cryoprotectants were then removed by ultrafiltration using Amicon Ultra centrifugal units. The Luc-LNP suspension (5 μg mRNA/mL) in PBS was then administered to mice (C57BL/6L mouse, female, 6 weeks; Japan SLC, Inc.) via the tail vein. The injection volume was adjusted for body weight (10 μL/g). At 3 hours post intravenous injection, the luciferase activity in the liver was evaluated.

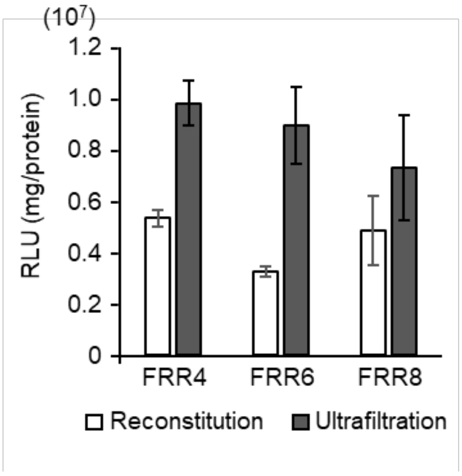

Among the all flow rate ratios (FRR) tested, the removal of the cryoprotectants by ultrafiltration improved the hepatic gene expression. The increase in the transfection activity was 1.83-fold, 2.73-fold, and 1.50-fold for FRR4, FRR6, and FFR8, respectively. It has been reported that the hypertonic condition is one cause of integrated stress responses which potentially link to the inhibition of translation[1]. Thus, in animal experiment, it is important to evaluate the activity after solvent replacement or dilution with saline when the formulation contains an additive such as a cryoprotectant.
